# Incorporating the Concept of a True Horizontal Reference in Dental Rehabilitation: A Novel Approach to Clinician‐Technician Communication

**DOI:** 10.1111/jerd.70188

**Published:** 2026-05-28

**Authors:** Alberto Rosmaninho, Gabriela Almeida, Eurípedes Vedovato, Rui I. Falacho

**Affiliations:** ^1^ Private Practice Oporto Portugal; ^2^ Institute of Implantology and Prosthodontics, Faculty of Medicine University of Coimbra Coimbra Portugal; ^3^ Private Practice São Paulo Brazil; ^4^ Department of Preventive and Restorative Sciences University of Pennsylvania School of Dental Medicine Philadelphia Pennsylvania USA; ^5^ ADEMA University School, University of the Balearic Islands (UIB) Palma Spain; ^6^ Center for Innovation and Research in Oral Sciences (CIROS), Faculty of Medicine, University of Coimbra Coimbra Portugal

**Keywords:** communication, computer‐aided design, digital dentistry, facial planes, natural head position

## Abstract

**Objective:**

Describe an accessible method for incorporating a horizontal reference line directly into a frontal facial photograph, thereby allowing the transfer of the patient's natural head position into a digital workflow without the use of additional positioning devices or specialized equipment.

**Clinical Considerations:**

The treatment planning of a male patient undergoing esthetic rehabilitation was conducted according to two distinct techniques. The novel strategy to establish a horizontal line, in which the line is directly placed behind the patient during the initial photographic record was compared to the usually employed approach in which the horizontal reference is based on the interpupillary line. Different mock‐ups were obtained allowing a thorough detection of the differences between techniques and the consequent treatment implications.

**Conclusions:**

This technique provides a standardized, accessible alternative to establish a horizontal reference that uses routine clinical photographs without additional hardware or complex setup.

**Clinical Significance:**

The horizontal reference line incorporated into the patient's photograph poses as an immediate visual validation of correct leveling and alignment. By providing a reliable orientation guide, it prevents positioning‐related inaccuracies, supports more consistent clinical decision‐making, and ultimately increases treatment predictability, which is particularly advantageous in complex cases.

## 1 Introduction

Contemporary dentistry has progressively shifted toward the use of digital workflows, with routine incorporation of photography, intraoral digitalization, and computer‐aided design and manufacturing technologies into daily clinical practice [1].

These tools have significantly enhanced the predictability of complex oral rehabilitations by allowing more precise planning and improved communication between clinicians and dental technicians. However, as treatment workflows become increasingly digital, the accuracy with which clinical information, particularly spatial orientation, is transferred from the patient to the virtual environment becomes critically important [1].

The esthetic perception of a smile is influenced by multiple interrelated factors, including tooth shape, proportion, color, symmetry, and their integration with the surrounding gingival tissues, lip position and the patient's overall facial characteristics [2, 3]. Achieving harmony between the rehabilitated dentition and the patient's facial appearance requires the accurate integration of facial reference planes during diagnosis and treatment planning, especially in cases involving extensive prosthetic esthetic or/and functional rehabilitation [2, 4, 5]. Even minor deviations from horizontal or vertical reference planes may be perceptible to the human eye, which is capable of perceiving deviations from parallelism with a sensitivity of approximately one degree [2]. Consequently, inaccurate perception or small errors in spatial orientation can result in clinically relevant esthetic inaccuracies [5].

One of the major challenges associated with digital workflows lies in the absence of inherent facial and spatial context within virtual models. Three‐dimensional datasets/digital dental models are frequently evaluated in isolation, without reliable reference to the patient's true head orientation. To address this limitation, both intracranial and extracranial reference systems have been proposed [6]. Intracranial references, such as the interpupillary or commissural lines, are widely used due to their accessibility; however, their reliability may be compromised by facial asymmetries, anatomical variability, or deviations in head posture [2, 5, 6, 7]. In contrast, extracranial references based on the natural head position (NHP) rely on true horizontal planes and have demonstrated greater reproducibility for facial analysis and esthetic planning, as well as for transferring the information and allowing dental technicians to work on a virtual patient [6, 7].

Several techniques have been described to record and transfer the natural head position into the digital environment, including two‐dimensional photography, radiographic methods, as well as three‐dimensional imaging systems [7, 8, 9]. Although effective, many of these approaches require dedicated external devices or positioning systems that may not be readily available in routine clinical practice and may interfere with the patient's ability to maintain a relaxed and natural head posture.

Over time, digital photography has become a standard and widely accessible diagnostic tool, enabling more accurate diagnosis, detailed esthetic and functional analysis and facilitating communication between clinicians and dental technicians [10]. Frontal facial photography, in particular, offers a practical method to capture the patient's natural head position [7, 8, 9]. Nevertheless, practical obstacles such as camera tilt, inconsistent positioning, or visual distortions with digital planning software may alter the perceived horizontal plane reference, potentially leading to misinterpretation during virtual alignment.

The purpose of this clinical technique paper is to describe a simple and accessible method for incorporating a horizontal reference line directly into a frontal facial photograph, thereby allowing the transfer of the patient's natural head position into a digital workflow without the use of additional positioning devices or specialized equipment. This technique is intended to serve as a qualitative orientation aid that enhances clinician–technician communication and improves the spatial orientation of virtual models during esthetic rehabilitation planning.

## 2 Technique Description

**FIGURE 1:**
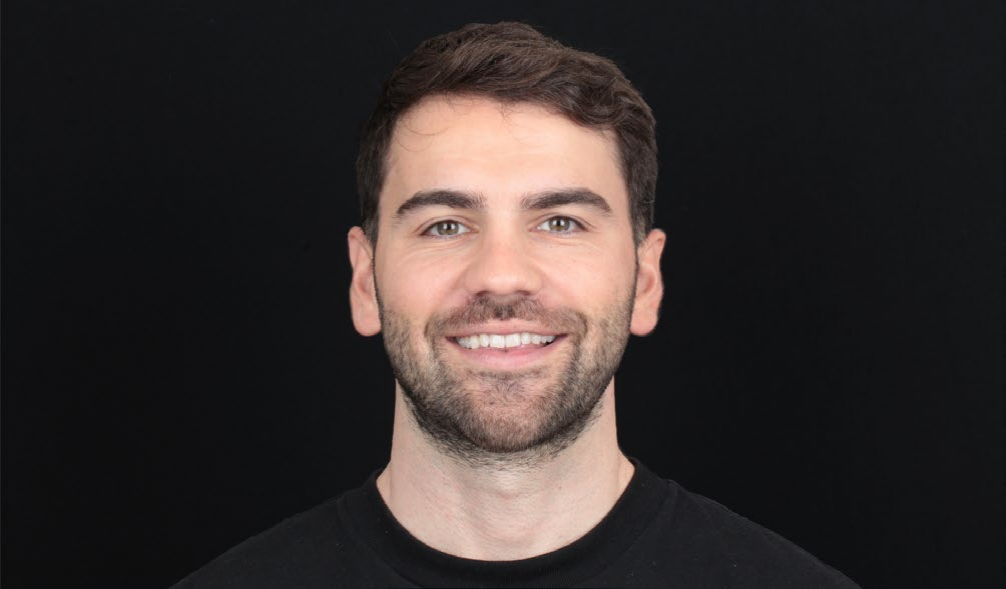
Extraoral frontal photograph of the patient.

A male patient expressing the desire to undergo an esthetic rehabilitation of the anterior sector, due to dissatisfaction with the shape and size of his teeth, underwent comprehensive diagnostic data collection (Figure 1). The records were obtained using the technique under description in this article. In parallel, a conventional strategy was also applied to the same patient in order to allow direct comparison between both approaches.

In routine prosthetic rehabilitation planning, extraoral photographic records and intraoral conventional or digital impressions are among the data to be collected and then sent to the dental laboratory. Facial photographic records are used by dental technicians to position the three‐dimensional (3D) digital model of the maxillary arch, primarily by aligning the facial midline and occlusal plane [5].

**FIGURE 2:**
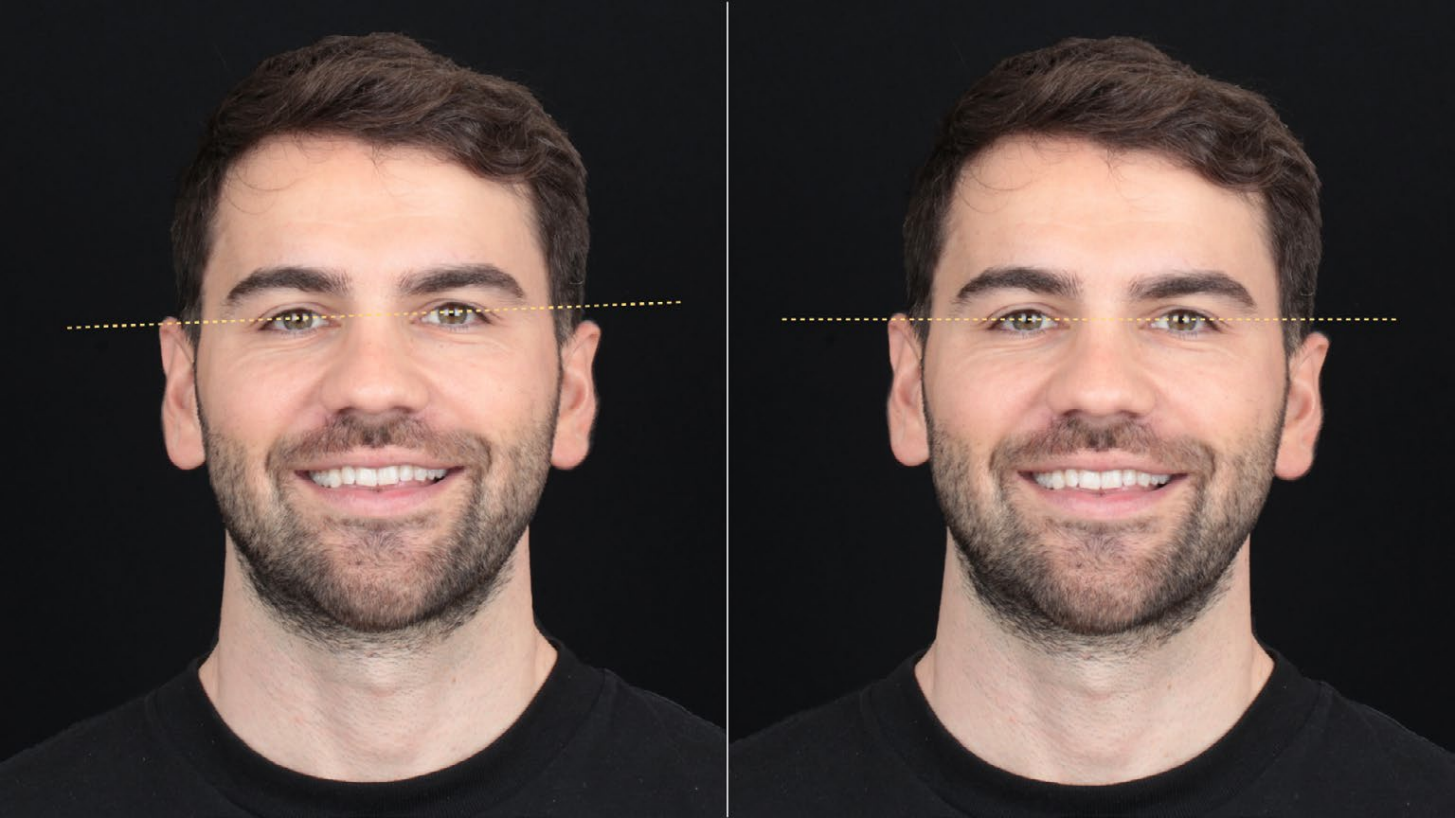
Alignment of the frontal photograph according to the IL.

**FIGURE 3:**
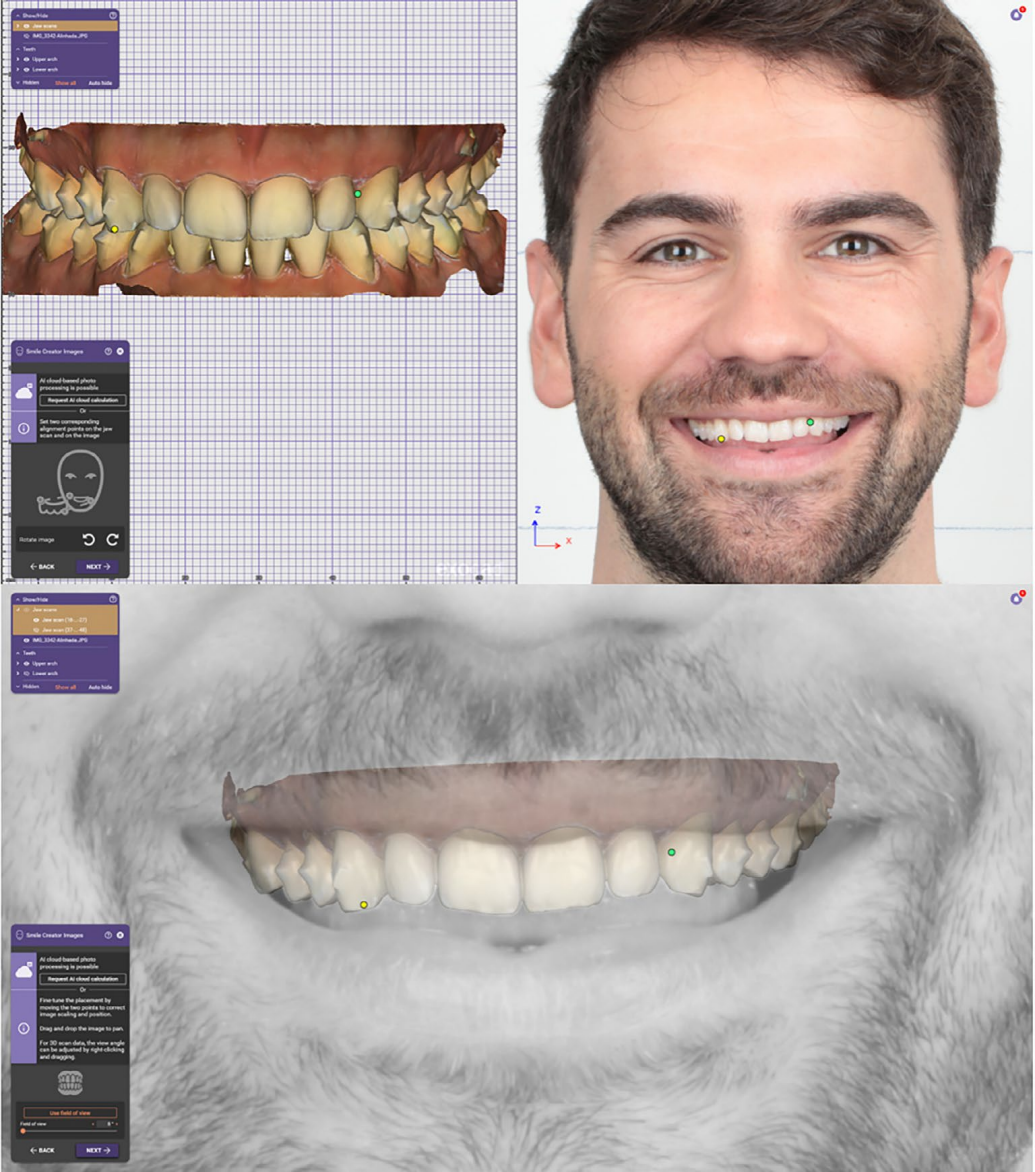
Alignment and superimposition of the frontal photograph and the STL file of the intraoral digitalization, according to the IL orientation.

In the absence of explicit spatial references, technicians usually rely on the interpupillary line (IL) to horizontally align the frontal facial photograph, as this reference is also suggested by most digital planning software interfaces (Figure 2). The aligned photograph is then used to superimpose the STL file obtained from the intraoral digitalization, allowing virtual mounting and subsequent computer‐aided design (CAD) procedures (Figure 3). However, it has been demonstrated that the interpupillary line may induce some errors, as it is influenced by anatomic facial asymmetries or posture deviations [2, 5, 6, 7].

**FIGURE 4:**
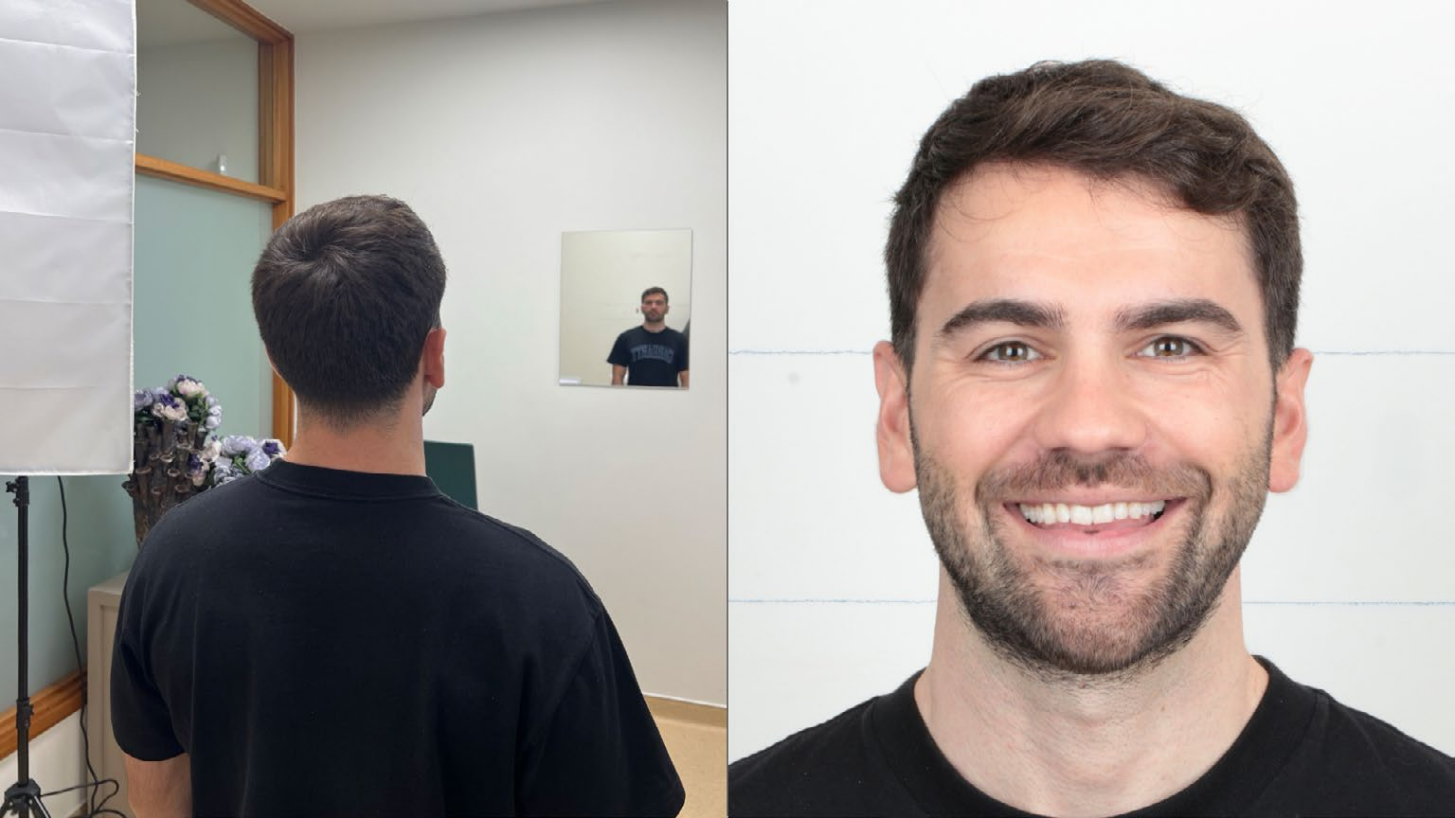
Frontal facial photograph incorporating the horizontal reference line, obtained with the patient in natural head position.

The proposed technique of this paper consists of incorporating a true horizontal reference (THR Technique) directly into the wall or background of the frontal facial photograph at the time of image acquisition. Prior to photography, a clearly visible horizontal line is drawn or marked on the wall or background behind the patient, resorting to a spirit level. The patient is then positioned as close as possible to the background wall in order to reduce the spatial discrepancy between the facial plane and the reference line, in natural head position by being instructed to focus on a distant point at eye level, typically by looking into a mirror placed in front of them, allowing spontaneous and relaxed head posture aligned with the true horizon [7, 8]. Facial photographs are subsequently taken respecting conventional photographic principles and ensuring that the horizontal reference line is fully visible within the frames for correct orientation of the patient position (Figure 4).

Frontal photographs should be obtained with the camera centered on the patient's facial midline, with the optical axis approximately perpendicular to the facial plane, and with consistent photographic settings whenever possible, including camera height, shooting distance, and focal length.

As the horizontal plane is defined by the THR, strict horizontal parallelism between the camera and the floor is not mandatory, as any camera tilt can be corrected during digital alignment using the reference line embedded in the image. This correction primarily applies to roll discrepancies (cant) visible in the frontal image and does not adequately control yaw or pitch deviations, which remain dependent on correct patient positioning and photographic technique during image acquisition.

**FIGURE 5:**
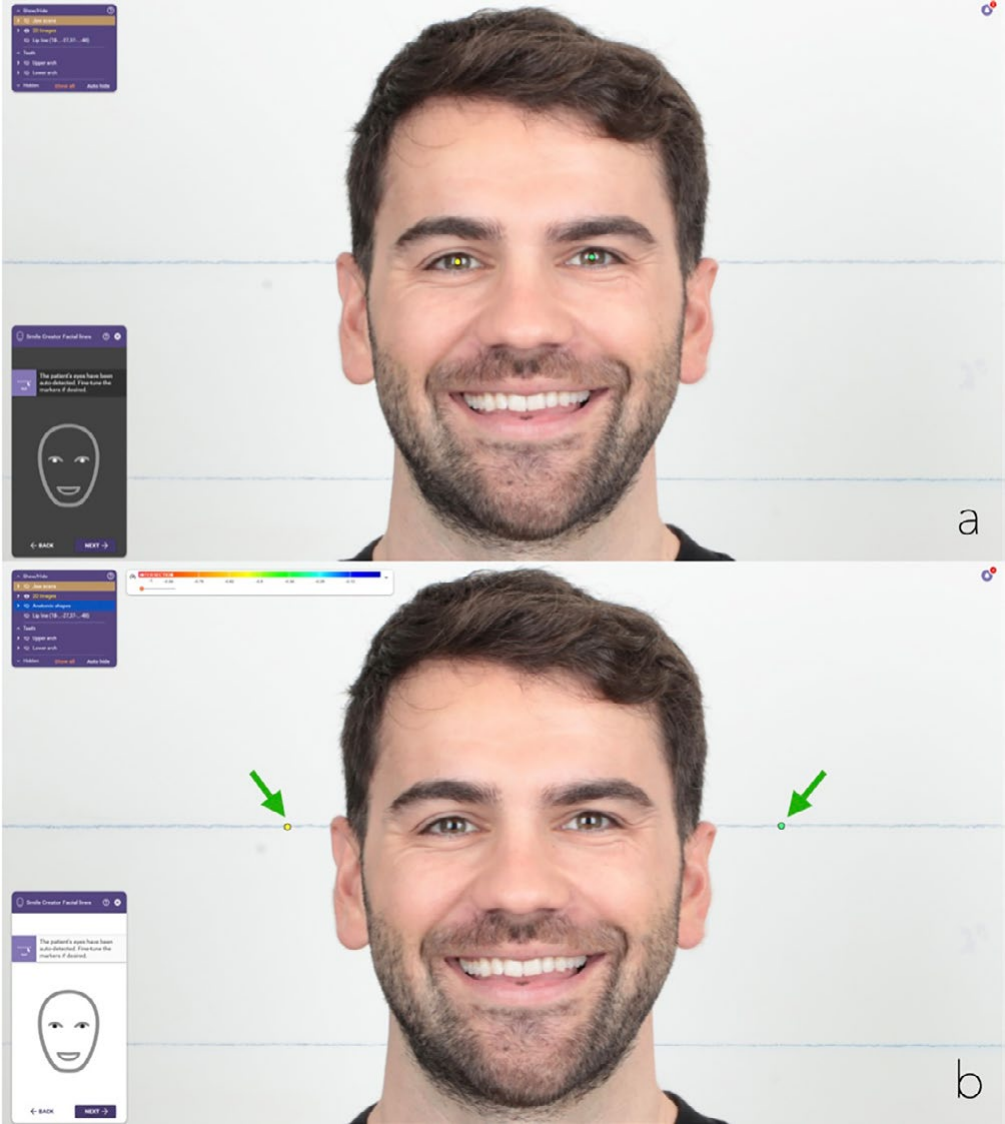
Difference in horizontal alignment of the frontal photograph depending on the reference used: (a) interpupillary line‐based alignment; (b) THR Technique.

**FIGURE 6:**
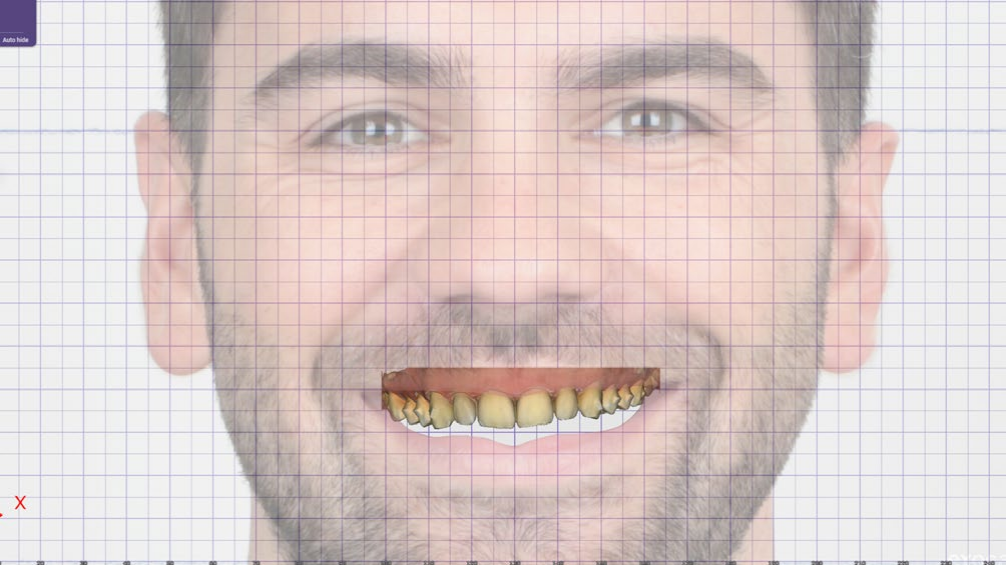
Alignment and superimposition of the frontal photograph and the STL file of the intraoral digitalization, according to the THR Technique.

Once transferred to the laboratory, the presence of the THR within the photograph provides the dental technician with an unequivocal reference for horizontal orientation. This allows the technician to align the photograph according to the true horizontal plane rather than relying on anatomical facial references alone. Additionally, the frontal photograph enables visualization of any discrepancy between the interpupillary line and the true horizontal plane. In the present clinical case, a difference of approximately 3° between the THR and the IL was observed, resulting in different horizontal alignments of the same photograph depending on the reference used (Figures 5 and 6). This angular discrepancy was obtained by digitally tracing the THR and the interpupillary line on the frontal facial photograph and measuring the angle formed between them using the planning software. This value should be interpreted as a descriptive clinical observation in the present case, rather than as the result of a formal validation or reproducibility analysis.

**FIGURE 7:**
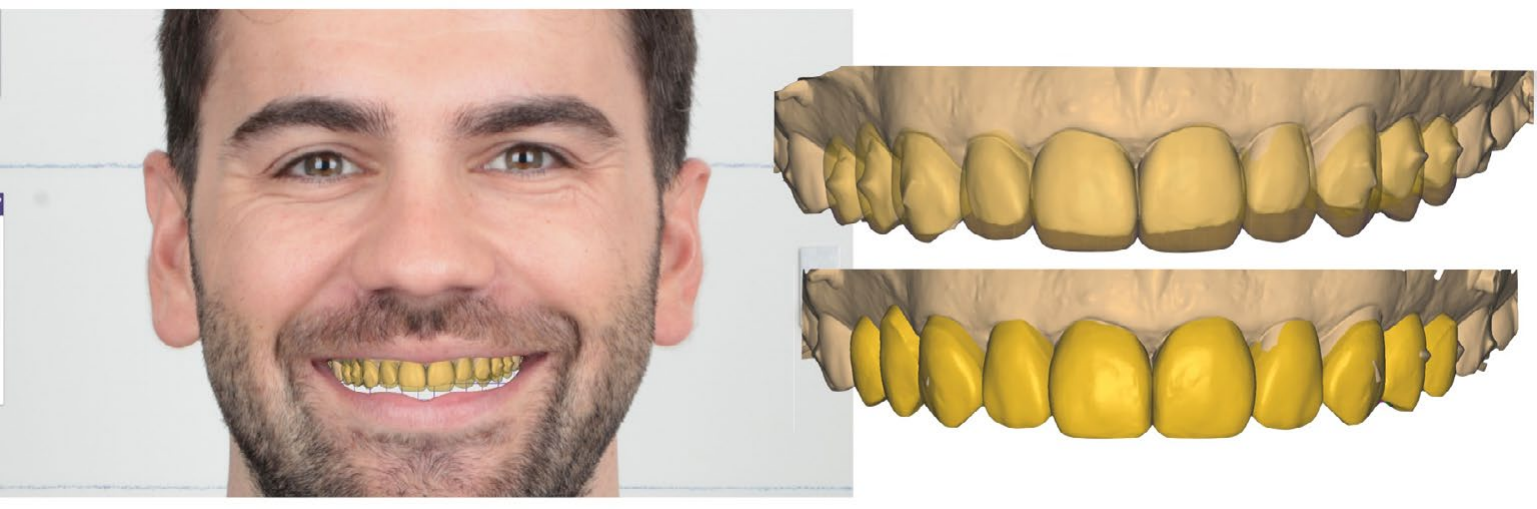
Alignment and digital wax‐up based on the THR Technique.

Alignment according to the THR Technique was applied to the present clinical case, in order to fabricate a digital wax‐up and respective mock‐up (Figure 7).

**FIGURE 8:**
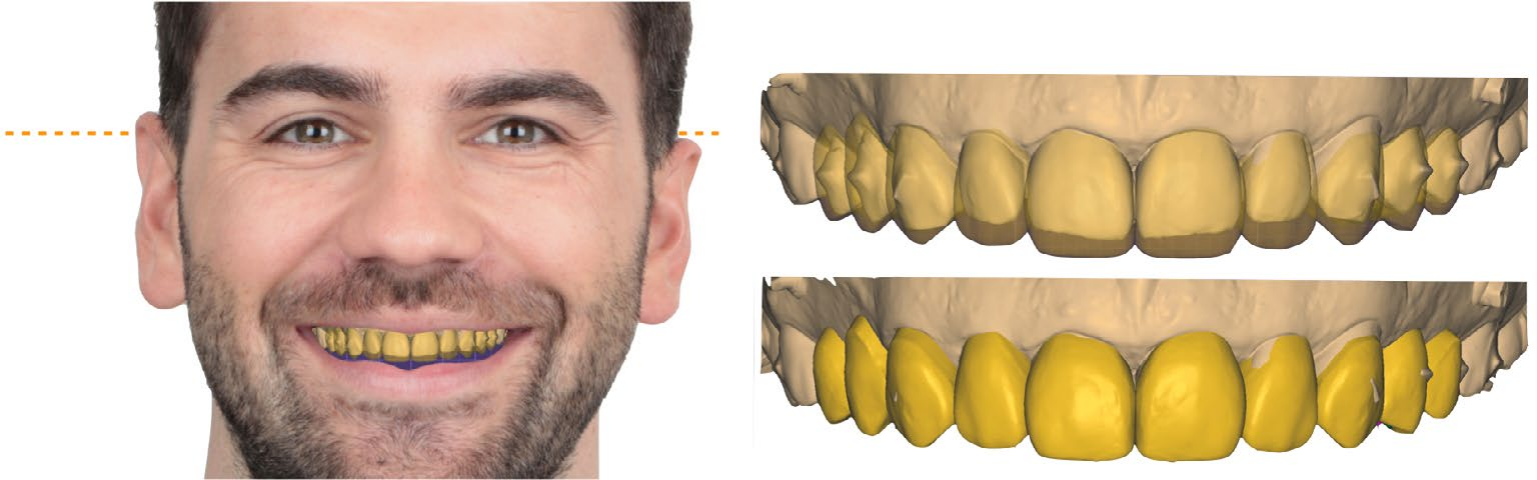
Alignment and digital wax‐up based on the interpupillary line.

Concomitantly, interpupillary line‐based alignment (Figure 8) was also applied to the same patient, and the same process of wax‐up and mock‐up was conducted.

**FIGURE 9:**
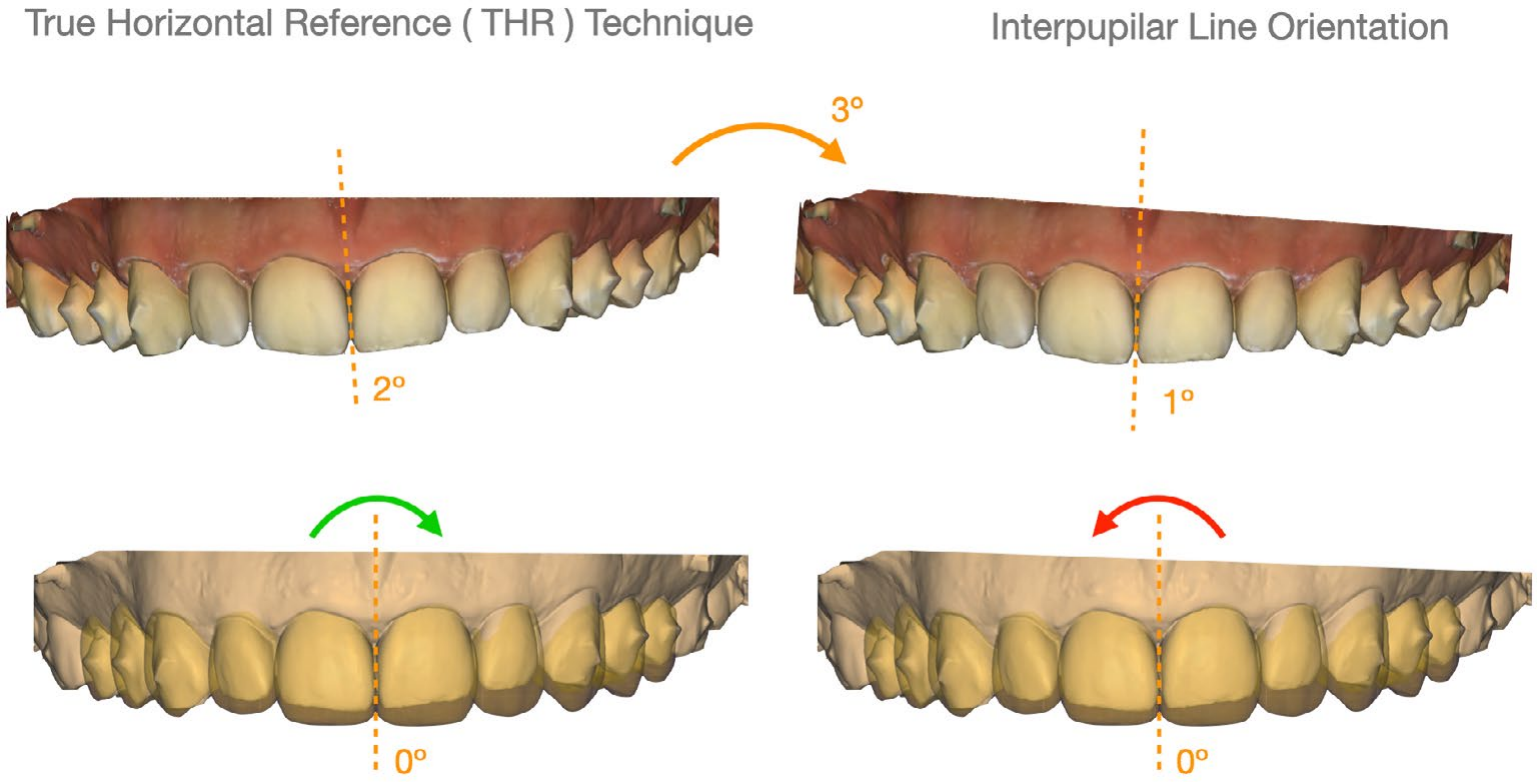
Comparison between the digital wax‐ups, resulting from the different alignments (THR Technique on the left and IL orientation on the right).

**FIGURE 10:**
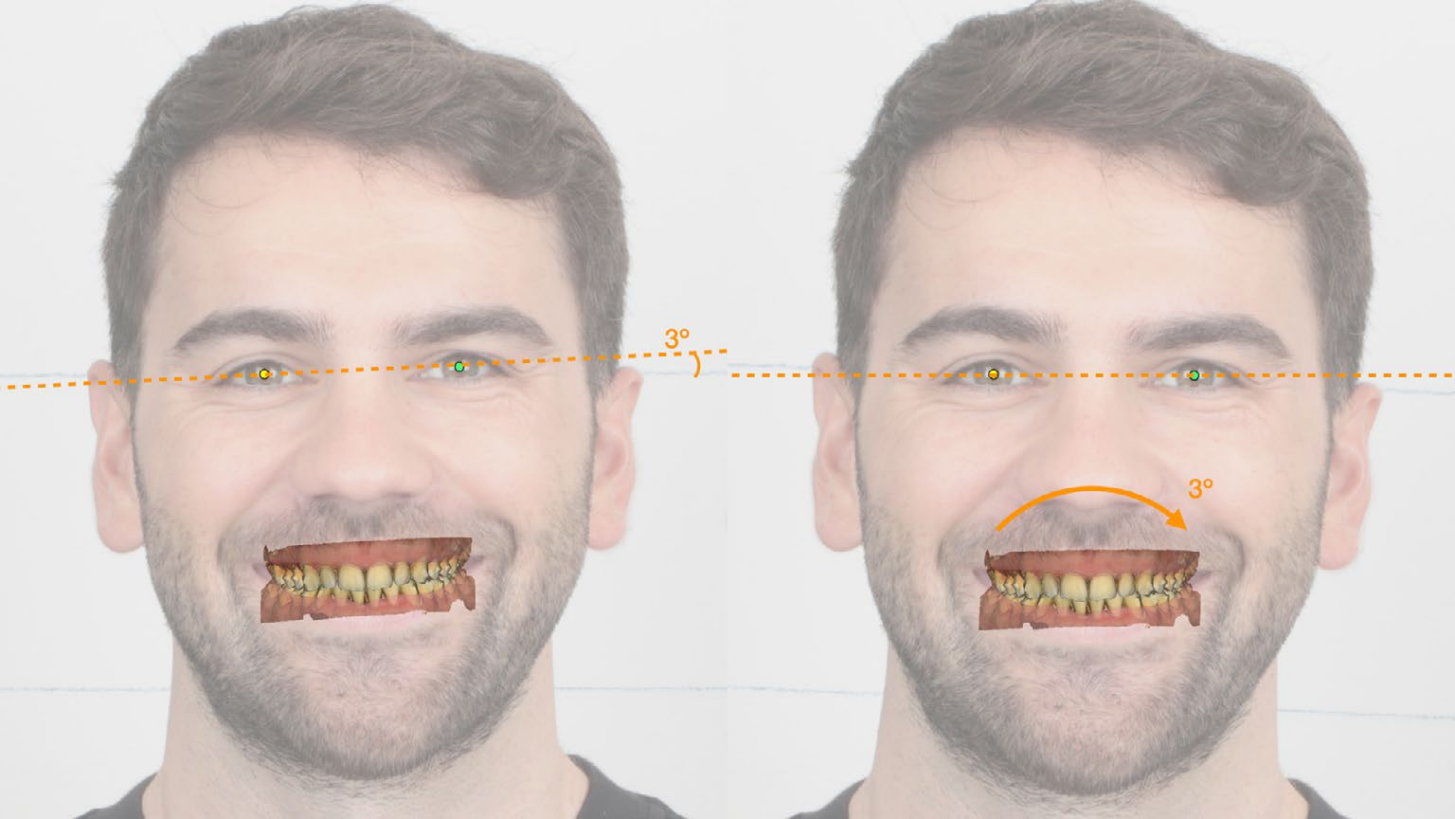
Discrepancies according to the different corrective strategies (THR Technique on the left and IL orientation on the right).

When the maxillary position was analyzed using the THR Technique, a 2‐degree axial inclination toward the first quadrant was identified. In contrast, alignment based on the interpupillary line resulted in an opposite inclination, with a 1‐degree deviation toward the second quadrant (Figure 9). Although the final wax‐ups appeared visually similar, the corrective strategies differed. Using the THR Technique, axial correction was achieved by inclining the dental axes toward the second quadrant, whereas interpupillary‐line alignment required inclination toward the first quadrant (Figure 10).

**FIGURE 11:**
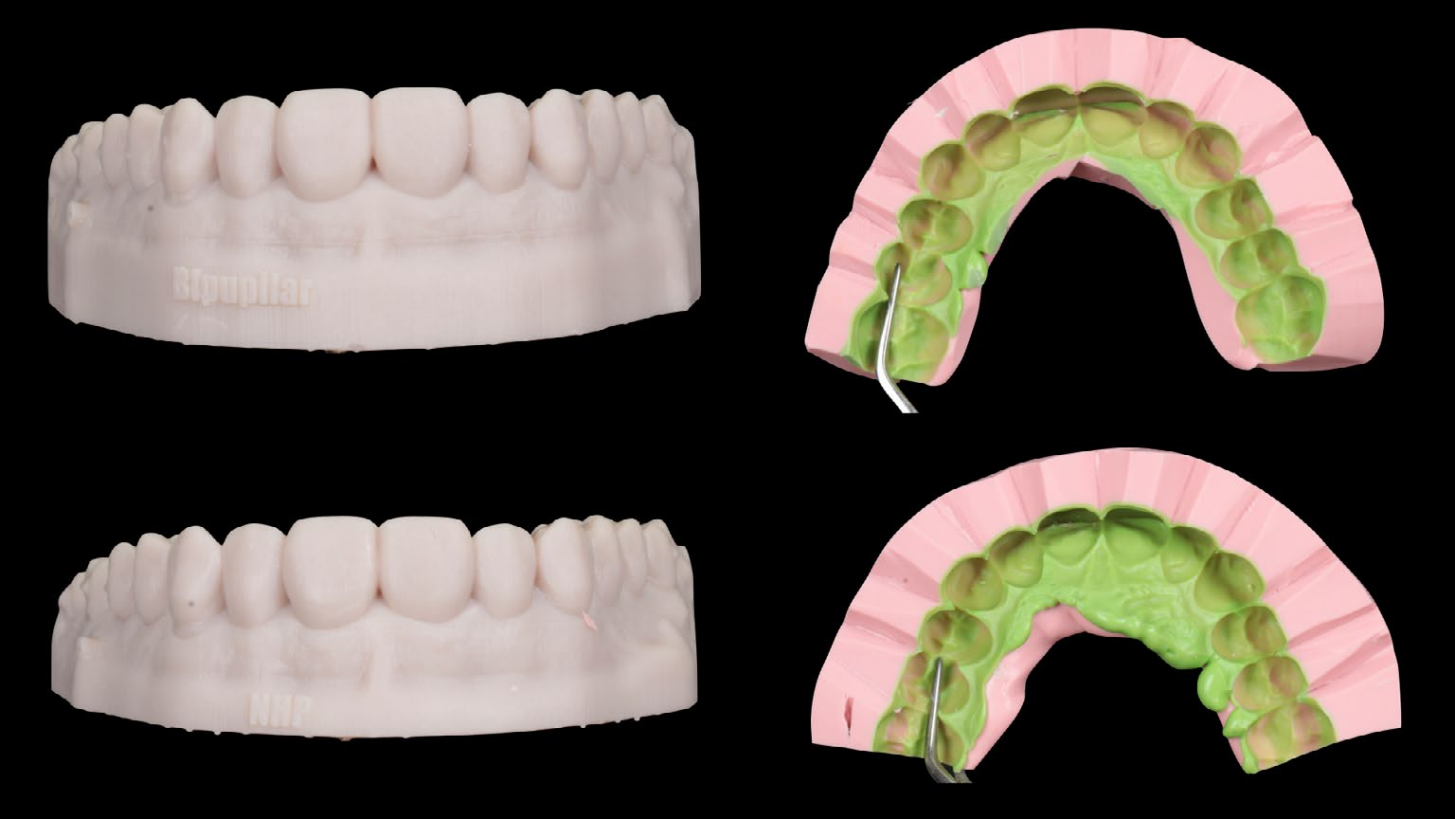
Distinct wax‐ups and respective mock‐up keys.

**FIGURE 12:**
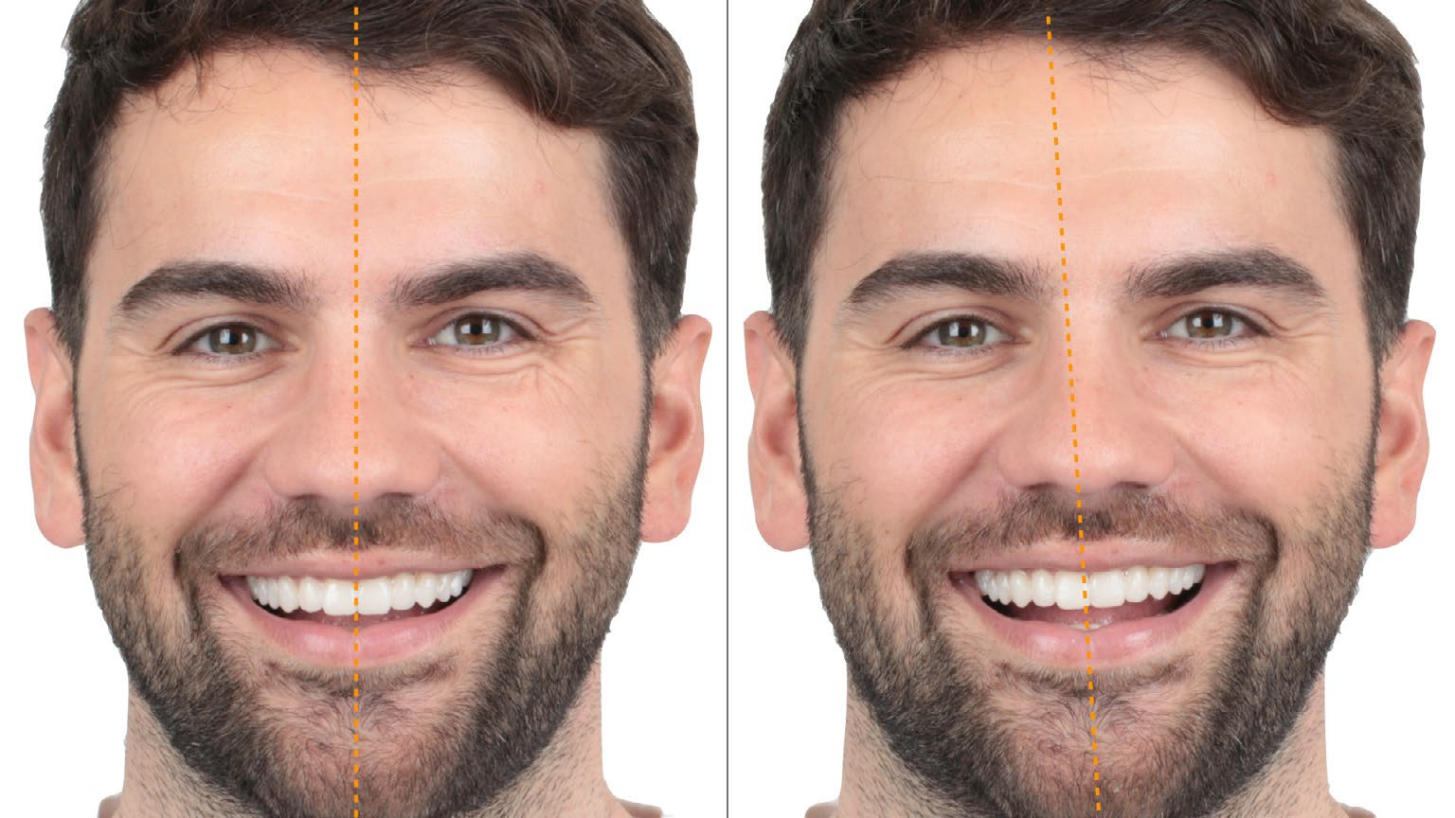
Intraoral mock‐up evaluation: THR Technique (left) and interpupillary line‐based alignment (right).

Given that THR Technique represented the correct maxillary orientation in this case, the interpupillary‐based correction not only failed to compensate for the initial discrepancy but amplified it. This error became clinically evident only during the intraoral mock‐up (Figure 11), when dental inclination could be assessed relative to the patient's natural head position (Figure 12).

When performing the mock‐up on the patient, the wax‐up generated using the THR Technique demonstrated an esthetic plane without inclination of the teeth long axis. In contrast, the wax‐up guided by the interpupillary line exhibited a visibly tilted esthetic plane, resulting in discrepancies in tooth length and axial inclination between the right and left quadrants.

This discrepancy occurred because the patient presented a 3° difference between the two lines. When alignment is performed using the interpupillary line, the digital planning software tilted the entire dataset (frontal photograph and maxillary STL), introducing a corresponding error in maxillary orientation. As a result, the diagnostic wax‐up was performed on a misaligned virtual model, leading to an incorrect occlusal plane and altered tooth axis orientation. Such discrepancies may remain undetected during the digital planning phase and only become evident during intraoral mock‐up evaluation.

## 3 Discussion

In esthetic and functional dental rehabilitation, the accurate communication of spatial orientation between the clinical and laboratory environments is essential to achieve predictable outcomes. Horizontal reference planes play a central role in this process, as deviations from parallelism, even of small magnitude, may be readily perceived and translated into clinically relevant esthetic discrepancies [2]. In this context, the ability to reliably convey the patient's true horizontal orientation to the dental technician becomes particularly important when digital workflows are employed.

More than 50% of the population presents facial asymmetries, inclinations or deviations that limit the reliability of conventional intracranial anatomical references [2] Consequently, the interpupillary line, despite its widespread use, does not consistently coincide with the true horizontal plane in all patients and has been reported to be an adequate reference in only a limited subset of cases [2, 5, 6, 7]. When such anatomical references are used without additional contextual information, the resulting digital alignment may inadvertently introduce systematic errors during virtual mounting and diagnostic wax‐up procedures.

When the mock‐up generated using the interpupillary line is evaluated against a black background without any external reference, the inclination error is difficult to perceive. This occurs because the absence of a true horizontal reference biases visual perception toward facial landmarks, such as the interpupillary line, which in this patient does not represent a reliable horizontal reference. Consequently, communication between the clinician and the technician becomes compromised, creating a gap between the clinical perception and the interpretation of the photographic image during the digital workflow. This discrepancy often leads to wax‐up corrections based on trial and error, as photographic misalignment and unreliable horizontal cues coexist.

Therefore, the presented technique emphasizes the incorporation of an external horizontal reference directly into the photographic record, allowing both clinicians and dental technicians to visualize the patient's natural head position relative to the true horizontal plane. By embedding the horizontal reference line within the image itself, the technique reduces reliance on subjective interpretation of facial anatomy and provides a clear and reproducible guide for digital alignment.

Furthermore, the use of a visible horizontal reference line, rather than a physical or digital leveling device during image acquisition, provides an additional means of verification that the photograph is correctly aligned, thereby improving reliability and safeguarding both clinician and technician in daily practice. This approach is particularly relevant in digital planning environments, where visual cues such as software background grids or reference axes may accentuate perceived asymmetries and prompt inadvertent corrective adjustments by the dental technician.

In the clinical example presented, a discrepancy of approximately 3° between the interpupillary line and the horizontal reference line resulted in a measurable alteration of maxillary orientation when the interpupillary line was used as the sole reference. This misalignment propagated throughout the digital workflow, affecting the occlusal plane and the axial inclination of the anterior teeth, and only became evident during intraoral mock‐up evaluation. In contrast, alignment based on the horizontal reference line allowed preservation of the patient's true head orientation, resulting in a more harmonious esthetic plane and improved symmetry between the right and left quadrants.

Several devices and techniques have been proposed to transfer the natural head position into digital workflows [4, 7, 8, 9, 11, 12, 13, 14]. However, many of these approaches require dedicated equipment or physical positioning aids that may not be readily available in all clinical settings and may interfere with the patient's ability to maintain a relaxed and natural posture. The alternative of aligning the camera itself using a leveling device can also be challenging in a clinical setting, as accurately elevating a tripod to eye level and maintaining a static setup can be technically difficult.

The technique described in this article offers a standardized, accessible and simplified alternative by leveraging standard photographic records already routinely obtained in clinical practice, without the need for additional hardware or complex setup. The presence of a horizontal line within the image serves as visual proof for both clinician and technician that the image is properly leveled, diminishing the probability of clinical errors and improving outcome predictability, particularly when conducting complex and extensive functional and esthetic rehabilitations.

Some limitations of this technique should be acknowledged. The accuracy of the horizontal reference depends on correct leveling, placement, and visibility of the line within the photographic frame, as well as on the patient's ability to assume a natural head position during image acquisition. Furthermore, although the embedded horizontal reference improves control of roll discrepancies in frontal photographs and those are exactly the aim of this technique, it is less effective in controlling yaw or pitch deviations, which remain inherent limitations of two‐dimensional image capture. In addition, perspective distortion and parallax may still influence perceived orientation, particularly because the facial plane and the background reference line are not coincident in space. This protocol can also be affected by improper photographic technique and still requires manual alignment of a three‐dimensional maxillary STL dataset with the facial photograph. Finally, this approach does not provide quantitative validation of horizontal accuracy and should therefore be interpreted as a qualitative aid rather than a measurement tool. Future studies incorporating quantitative assessments or comparative analyses with established natural head position recording systems may help further clarify the precision and reproducibility of this method.

## 4 Conclusion

The proposed method relies on the inclusion of a horizontal reference line within the photograph of the patient for visual verification of correct alignment, thereby minimizing clinical inaccuracies and increasing reliability, particularly in demanding rehabilitation cases. Additionally, it offers a standardized and easy solution, as it eliminates the need for extra equipment or intricate configurations. Therefore, this approach is designed to function as a supportive clinical communication and orientation tool, strengthening collaboration between clinicians and technicians and improving consistency in the three‐dimensional positioning of digital models throughout esthetic treatment planning.

## Funding

The authors have nothing to report.

## Disclosure

The authors have nothing to report.

## Conflicts of Interest

The authors declare no conflicts of interest.

## Data Availability

The data that support the findings of this study are available from the corresponding author upon reasonable request.
